# Power distance within online and face‐to‐face medical education in Sri Lanka and the UK

**DOI:** 10.1111/medu.70025

**Published:** 2025-09-17

**Authors:** Amaya Ellawala, Alison Ledger, Harith Wickramasekara

**Affiliations:** ^1^ Health Professions Education Unit, Hull York Medical School University of York York UK; ^2^ Academy for Medical Education, Faculty of Health, Medicine and Behavioural Sciences University of Queensland, Herston campus Brisbane Australia; ^3^ Department of Medical Education, Faculty of Medicine University of Kelaniya Kelaniya Sri Lanka

## Abstract

**Introduction:**

The student‐teacher relationship can impact learning ‐ power distance is an integral component of this relationship. This study drew on Hofstede's Model of National Culture to compare UK and Sri Lankan students' and teachers' experiences of power in online and face‐to‐face learning environments.

**Methods:**

A qualitative, exploratory approach was employed. Fourteen interviews and two focus groups were undertaken with undergraduate medical students and teachers in the two settings, during which participants drew their perceptions of power in both learning environments (online and face‐to‐face). These rich pictures were analysed using aesthetic analysis alongside participants' interview responses, to explore patterns and construct themes for reporting.

**Results:**

Though differences between cultures and learning environments were expected, teachers and students in both countries shared understandings of power distance in the teacher‐student relationship and expected the teacher to hold power in both online and face‐to‐face environments. Teachers expressed a desire to lessen hierarchical relationships and attempted to minimise power differentials when online or face‐to‐face. Strategies for reducing power distance included addressing students by name, using informal and respectful communication, establishing common ground and showing their ‘humane’ side.

**Discussion:**

To achieve greater partnership with students, it is recommended that educators recognise students' strengths and leverage possibilities within their chosen learning environment to modulate the degree of power distance, promote participation and optimise learning.

## Abstract 2

Mind the gap! This study based in Sri Lanka and the UK explores how student‐teacher power distance is perceived in remote and face‐to‐face educational settings along with how it influences learning.

## 1 INTRODUCTION

The student‐teacher relationship is recognised as an important facet of the undergraduate medical learning environment, with an influence on learning. Elements of this relationship, such as communication, rapport and concern for learner wellbeing, are reported as important.^1^, ^2^, ^3^ Power distance is an integral component of the educational relationship. Traditional dynamics of power between teacher and student, in which the teacher is more powerful, can positively influence acquisition of knowledge and learner self‐esteem,^4^ and can enable students to practice safely in a clinical environment.^5^ Conversely, traditional power dynamics can inhibit student engagement and collaborative learning.^4^, ^6^

Hofstede refers to power distance as an entity encompassing human inequality and how individuals respond to those in positions superior or inferior to themselves.^7^, ^8^ Hofstede described this phenomenon as one dimension of his 6‐D Model of National Culture, which outlines specific dimensions that can be used to describe varying cultures from around the globe.^7^, ^8^ According to this theory, within high‐power distance societies (e.g., some Asian countries such as Sri Lanka), societal hierarchy based on position, authority and age is more readily accepted and respected than in low‐power distance contexts (e.g., UK). Hofstede describes how power distance in teacher‐student relationships can reflect the hierarchy of the encompassing culture, where in hierarchical societies, there can be a more pronounced power distance between the learner and educator.^9^

Several studies have explored power distance within student‐teacher relationships in higher education, reporting both wide and narrow power gaps either in congruence with the broader society or in contrast with them.^10^, ^11^, ^12^, ^13^ Hierarchy within clinical learning environments is well documented.^14^, ^15^, ^16^ However, literature on power distance in classroom‐based medical training is sparse. One study in Saudi Arabia found conflicting views on power distance, with medical students perceiving larger gaps between student and teacher than their teachers.^17^ Another study conducted in the USA reported strong perceptions of hierarchy within undergraduate training and beyond.^18^

Much of this previous work looks at power distance in the face‐to‐face learning environment, which until recently served as the predominant mode of learning in primary degree programs, including medicine. However, the 2020 global pandemic instantaneously thrust most of higher education into remote learning. More than five years later, many programmes continue to offer hybrid learning opportunities. Although remote learning has been in use for several decades, it has, without doubt, witnessed exponential growth within the recent past. This is particularly pertinent within medical training programmes, where areas previously not considered feasible for remote learning, were forced to adapt to online formats.

As online learning has become a significant component of medical training, it is imperative that we understand more about educational relationships within this environment. Overall, evidence related to teacher‐student power distance in remote higher education is limited. Zhang interviewed US‐based Chinese undergraduate students in a range of disciplines (excluding medicine) about their perceptions of teacher‐student power distance and reported highly hierarchical relationships in the online environment.^19^ Suwinyattichaiporn et al, administered a collection of inventories to measure student‐teacher interactions and Facebook activity and discovered that more frequent teacher‐student interactions on Facebook led to lower perceptions of power distance.^20^ Notably, this study was undertaken in a university in Thailand, a country typically regarded as having high power distance. A comparison of Chinese and British postgraduate student‐tutor relations, using questionnaires and interviews, revealed more informal and less hierarchical relationships in the high power‐distance Chinese context when compared with the low power‐distance British.^21^ Collectively, these studies reveal that perceptions of hierarchy within remote educational relationships are not always reflective of the power distance in the societies within which they are situated. However, none provide information related to medical training specifically.

Online learning is likely to remain a mainstay of medical education and is essential in medical schools that serve rural and remote communities. Therefore, we must strive to appreciate the nuances of the learning format and how it impacts the student‐teacher relationship. As medical educators based in Sri Lanka and the UK (and one having worked in both countries), we were curious as to how the different power‐distance dispositions of the two contexts (SL – high power distance, UK – low power distance) might manifest within undergraduate medical education, and particularly within online and in‐person learning environments. We therefore sought to answer the following questions: How is power understood within Sri Lankan and UK medical student‐teacher relationships? Does power distance between undergraduate medical students and teachers differ in the online and face‐to‐face learning environments? Do these perceptions vary between the contexts of Sri Lanka and the UK?

## 2 METHODS

We employed a qualitative, exploratory approach. Third and fourth‐year medical students, and teachers with over three years of experience in undergraduate medical education at the Universities of Kelaniya (Sri Lanka) and Leeds (UK) were recruited through purposive sampling complemented by snowballing. We approached staff and students known to have an interest in medical education research (e.g., UK students undertaking an intercalated degree in medical education), as we considered them more likely to volunteer during the COVID‐19 pandemic and to have previously reflected on student‐teacher power dynamics. Participants took part in interviews or focus group discussions online, as data collection occurred when in‐person interactions were restricted. Interviews lasted between 40 and 60 minutes, and focus group discussions between 60 and 90 minutes, during which perceptions related to teacher‐student power distance in online and face‐to‐face educational settings were explored.

Given the complex nature of the topic under exploration, we presumed that participants might struggle to express their perceptions of power distance. We therefore used rich pictures to facilitate expression. Rich pictures are ‘pictorial representations that attempt to capture an individual's perspective of a situation, including objects, ideas, people, character, feelings, conflicts and prejudices’.^22,p. 714–715^ They are a recognised method of eliciting perceptions around complex situations and gaining insight into less explicit aspects of an area under exploration,^23^ by serving as a starting point for articulating thoughts that are difficult to express.^24^ At the outset, participants were asked to share their understanding of power distance, after which Hofstede's definition of the term was shared with them. They were then asked to produce two drawings – one depicting their perceptions of power distance in the online learning environment and the other related to the in‐person environment. Participants were then asked to describe their drawings and any attached meanings. The interviewer (AE or HW) then posed probing questions for a deeper exploration of perceptions. For example, we asked some participants to explain why figures were drawn in differing sizes. Recognising that discussion is a platform for co‐construction of data between the interviewer and participant, participants were encouraged to edit their drawings where required, and interviews continued until the participant indicated they had conveyed all they wished to share.^24^

Previous studies using rich pictures have been conducted primarily in‐person.^25^, ^26^, ^27^ As this study was conducted exclusively online, some adaptations had to be made to the process of drawing. For instance, participants were instructed to have paper and drawing material to hand for the interview/focus group discussion – as the drawings were reliant on resources available to participants, they were mainly monochromatic.

Two separate but complementary approaches were taken to data analysis. Conversations were recorded and transcribed verbatim. Transcript data was coded inductively and then codes were brought together to develop shared‐meaning‐based interpretive themes.^28^ AE first coded all transcripts. HW coded a sample of transcripts, following which initial codes were reviewed and refined by AE. AE then developed initial themes, which were reviewed by AL as an additional measure to ensure clarity and coherence. AE and AL collaboratively finalised and named the final themes.

Participant drawings were analysed separately. Christancho and Hemlich describe two approaches to the analysis of rich pictures – a. where drawings are only considered a stimulus for conversation and not analysed separately and b. where drawings undergo aesthetic analysis along with separate analysis of interview transcripts.^24^ Recognising the richness of meaning to be derived from drawings, we opted for the second option. However, once again owing to the remote nature of the study and the wide geographical distribution of the research team, the method of analysis had to be adapted. Typically, aesthetic analysis has occurred in two parts^24^:

1. Rich picture viewing session – here, individual drawings are viewed in detail, first describing and then interpreting each in turn. Relevant interview content is then added to develop the interpretation further.
2. Gallery walk ‐ diverse stakeholders including the research team and participants are invited to collectively view several or all drawings, with a view to searching for patterns, similarities and differences across the collection.

We adapted these steps in the following ways:

1. Rich picture viewing session – a. drawings were shared through an online platform, where the research team described and interpreted drawings using written comments. Guiding prompts such as ‘What illustrative figures do you see?’ and ‘What metaphors do you see?’ were used to focus analysis. b. interview content was then added to the drawings, and a second round of interpretation was conducted.
2. Gallery walks – did not occur as a separate step but were incorporated into the above, by asking participants to look for patterns, similarities and differences across the set of drawings as a final step in analysis.

### 2.1 Reflexivity

All members of the research team are medical educators and worked at either of the two study locations during the research period. This study, therefore, was influenced by our insider researcher perspectives, particularly our own experience of learning to teach effectively online during the pandemic. Having worked in both study locations, AE had observed more hierarchical educational relationships in Sri Lanka compared to the UK and had also noted how digital communication served to diminish this hierarchy within the Sri Lankan context. There was an assumption, therefore, that the study findings might mirror these anecdotal experiences.

Ethics approval was granted by the Universities of Leeds and Kelaniya. As this was a study on student‐teacher power distance, we were cognisant of the potential limitations of students being interviewed by teachers. We aimed to minimise this impact by ensuring that interviews were conducted by members of the research team who had the least contact with students in the respective location. For example, AE had only taught the UK students as a guest lecturer on one occasion and was unlikely to be perceived as having influence on students' progression.

## 3 RESULTS

Twenty‐five participants (UK – 2 students [LS], 6 teachers [LT]; Sri Lanka – 11 students [KS], 6 teachers [KT]) were included through 14 interviews and 2 focus group discussions. Students were from different year groups, and teachers had at least 5 years of experience in medical education.

Through the combined analysis of transcript data and participant drawings, we constructed the following themes:

1. Power through different lenses
2. Displaying power
3. The dynamic nature of power distance
4. Narrowing the power distance in online learning

We will now discuss these themes in detail, illustrated with relevant quotes and drawings. Overall, when we compared Sri Lankan and UK contexts and face‐to‐face and online learning, there were more similarities than expected.

### 3.1 Power through different lenses

Power distance was most often described in terms of control of the learning environment, with recognition by teachers and students in both contexts that the teacher would often be the holder of power. Teachers used words such as *authority, control, stronger* or *leads* to describe this type of power. LT1 likened it to ‘*conducting an orchestra’*. Students described it in terms of hierarchy, dominance and inferiority.

“Power essentially implies that one party is stronger and has an authority or some sort of influence over the party, the weaker party”. (KT3)

“The teacher needs a level of dominance to keep the students in line, so that they can keep their attention on the lecture … if there is no dominance, then I don't think that there'll be any flow of information or flow of knowledge between the lecturer and the students”. (KS8)

**FIGURE 1:**
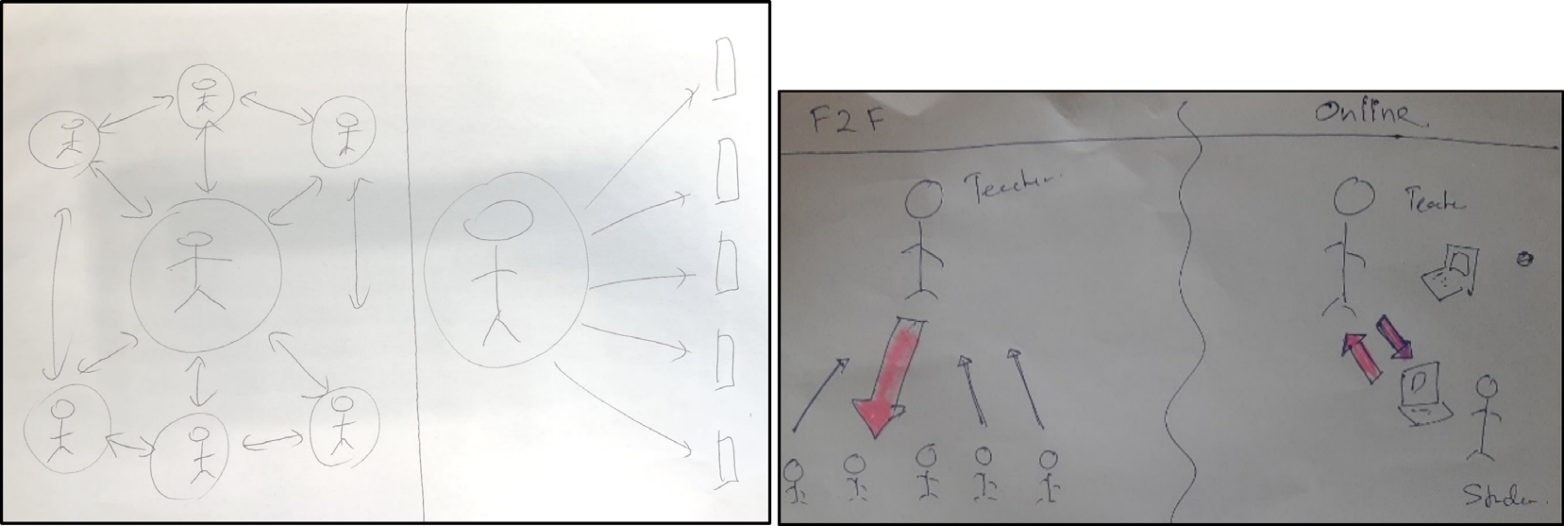
Power denoted through figure size and arrows (direction, size, colour) (LT5 and KT3). [Color figure can be viewed at wileyonlinelibrary.com]

We noted that in drawings, power was depicted through different figure sizes (with the teacher often larger than the student) and through symbols such as arrows illustrating the flow of power (see Figure 1).

**FIGURE 2:**
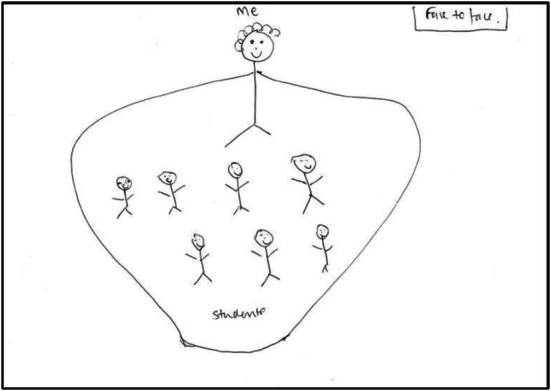
A ‘nurturing’ form of power (LT2).

However, there was an acknowledgement, again by both parties, that power should not be dominating and should not always lie with the teacher. For instance, LT1 talked of democratic power, LT2 described a ‘parental’ type of power (see Figure 2), whereas KS7 referred to an ‘inter‐connection’.

“For me, power distance doesn't necessarily mean it's just sort of taking and obey. There is sort of that two‐way street as well of being able to ask questions, being able to respectfully challenge if you've seen something differently”. (LS2)

It was evident, therefore, that power was viewed through different lenses, and that there was no singular conception of power unique to either context.

There was clear acceptance, however, that regardless of the mode of learning or geographical context, the teacher held power and therefore, a power gap existed between the student and teacher. This was indicated in drawings by the teacher constantly being drawn either apart, at a higher level, separated by barriers or larger than students. Teachers across both contexts spoke of the inherent nature of a teacher's power, which some argued did not change based on the mode of teaching.

“Obviously the teacher has more power because the teacher is the one given the power to conduct the session. And, we haven't changed as people. So, we are still us even if we are in an online environment or face‐to‐face environment, so those fundamentals don't change. It's just how it's perceived might change for some people.” (KT3)

Both parties felt that some gap in power was necessary for effective learning, but too great a power distance could have negative implications. UK participants reflected on how dynamics are changing with the consumerisation of learning, with students now wielding greater power.

“I don't think the hierarchy otherwise is quite as clear anymore as it used to be because they are the customer. And that has changed the power dynamic, in my view from before. And I have noticed that when I have been lent on after students complained and the likes where I feel my principles are being … .let's say pushed to the limit”. (LT6)

### 3.2 Displaying power

Power could take many forms. It was often described as being imbued in the teacher because of their role in facilitating learning and their responsibility for assessment and progression. The teacher was recognised as more powerful due to their comparative seniority, vaster expertise and experience. Power was also described in terms of the teacher's ability to influence punitive actions and, alternately, provide safeguarding for students. The role and speciality of the teacher were additional factors determining power. For example, some non‐clinical teachers perceived themselves as less powerful in the eyes of students than their clinical colleagues. Teachers felt that if their discipline was perceived as important by students, this allowed them more power in the classroom.

**FIGURE 3:**
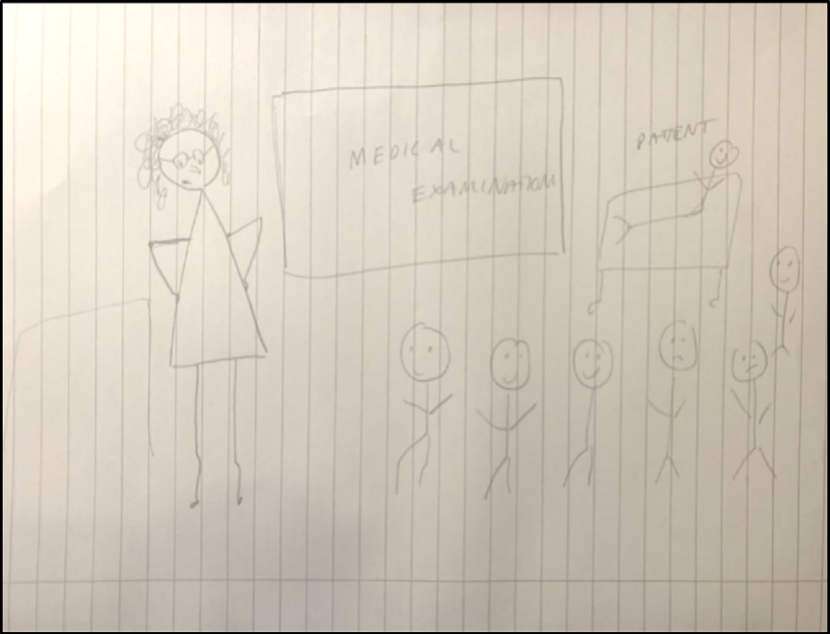
Power (in terms of being more knowledgeable) depicted through posture and spectacles (LS1). [Color figure can be viewed at wileyonlinelibrary.com]

Teachers were perceived to display power in a multitude of ways. Body language was one such manifestation. For example, postures such as hands‐on‐hips (see Figure 3) were associated with power in the face‐to‐face learning environment, whereas some used open body language to reduce any power gap with students. Other ways in which teachers would convey power were through an authoritative tone of voice, promotion of their expertise and use of titles such as Dr or Prof.

**FIGURE 4:**
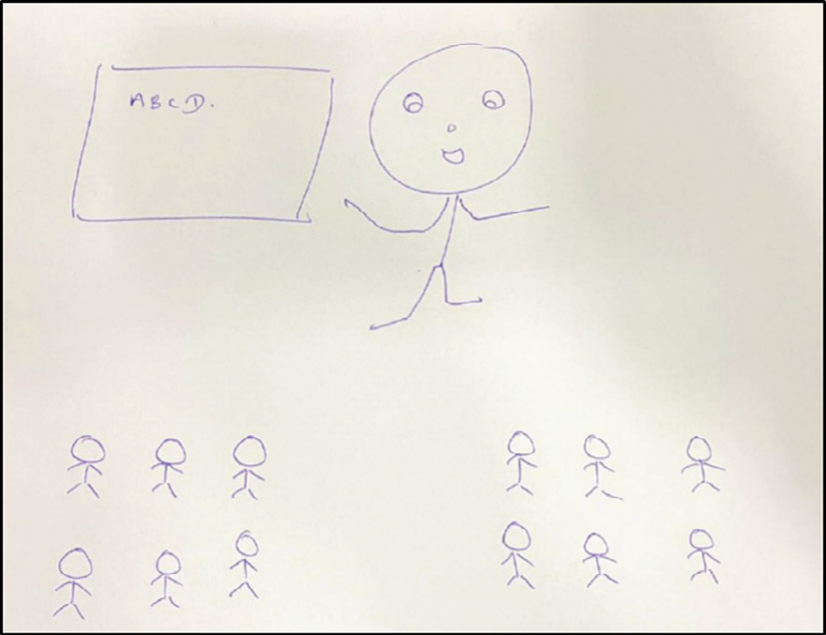
Teaching space arrangement and physical objects portraying teacher power (KT4). [Color figure can be viewed at wileyonlinelibrary.com]

Interestingly, use of physical objects like a lectern, or seating arrangement in the teaching space (e.g. theatre style vs. cabaret style, see Figure 4) was also seen as enabling or diminishing the power gap.

“I would purposely sit at the back of the room and not next to the screen. So I was distancing myself physically from the screen and the front of the room where quite often teachers stand because that's the power bit … .the computer, and the screen on the desk is the power bit” (LT1)

Many of these displays of power applied across in‐person and remote settings and in both geographical contexts, though participants did note the following difference in online settings.

“Their (teachers) body language is very relaxed and kind of reduces that power distance [online]” (LS2)

Some students felt that the face‐to‐face environment, where a more humanistic side of the teacher was visible, offered more opportunity for reducing power distance, though others described power associated with physical presence.

“The perception of power is more in the physical setting due to the physical presence”. (KS11)

### 3.3 The dynamic nature of power distance

It was observed that power was a constantly fluctuating entity and that power differences within an educational relationship were dynamic. Power was regarded as both a boon and a bane in both face‐to‐face and online settings.

“The teacher who communicates … who respects your intelligence and makes the classroom an open communicating place … . If you can openly express and discuss your ideas … that could translate as positive power. Punishing … that would be how power translates negatively. Sometimes the teacher who respects a student and who makes the more cooperative learning environment in reality would have more power over the students than a teacher who actually uses the negative aspects of it”. (KS11)

In both Sri Lanka and the UK, there was recognition that teachers and students made conscious efforts to either widen or narrow power gaps. Among teachers, the choice to either reduce or enhance power distance seemed to be a very personal one, and not dependent on cultural context. In general, teachers across both countries preferred to maintain a narrow power distance, while keeping some professional boundaries. It was felt that, at times, assertion of power was needed, for instance, when it was necessary to gain attention.

“I think it's a bit of a getting the right balance. I want the students to be comfortable and to feel at ease when they are with me in clinic and because that is very, very productive for their learning. But on the other hand maintain that element of professional distance so they would not behave with me like they behave with their peers”. (LT5)

“The power difference that exists at the moment in reality between students and teachers, it's still wide and doesn't need to be that. There is a definite power difference, although from the teacher's end we try not to maintain that at most times, but we also sometimes use that”. (KT3)

**FIGURE 5:**
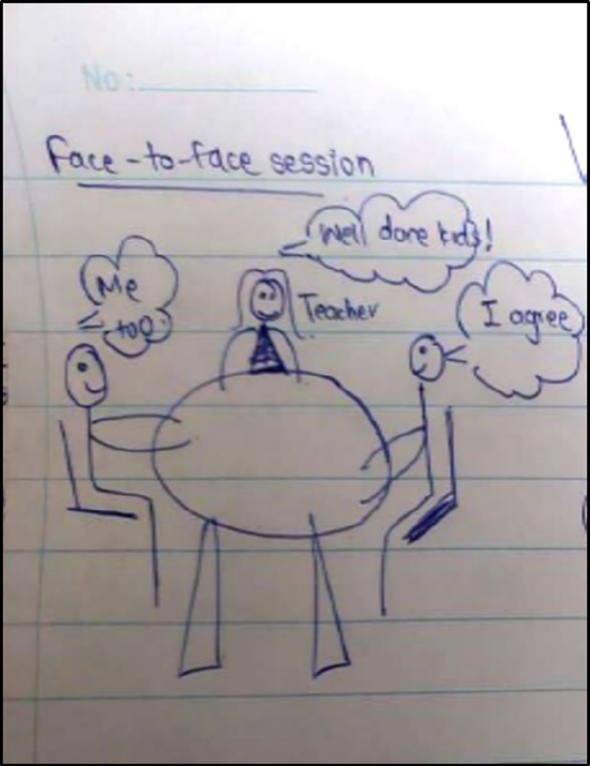
Creating a friendly atmosphere in the classroom (KS6). [Color figure can be viewed at wileyonlinelibrary.com]

Across both schools, some teachers favoured approaches like creating a friendly atmosphere in the physical classroom, positioning themselves among students rather than at the front of the class, addressing students by name, sharing personal anecdotes and enabling co‐creation with students to attempt a reduction in perception of power (see Figure 5). There was recognition that certain features of remote learning allowed discreet exertion of power.

“Online … I don't have to be so powerful. I can send a private message to an individual that is being disruptive and said, do you realize this is really not coming across very well without having to embarrass them in front of everybody else. If I'm in a classroom, I have to say this aloud. And maybe pinpoint them”. (LT6)

Teachers observed how students would sometimes reject attempts to work in partnership, and reinforce traditional hierarchical roles when learning clinical skills.

“I need my DOPS [directly observed procedural skills] to be signed off today, that's what I need from (you) today. I've come at them saying there are so many things you can learn by observing by doing. Have you got any specific learning needs? I have some potential targets we could aim for. Let's work together. They're saying actually I have very specific agendas and this is all I want to do. They're saying you are my teacher. I am your student. You have to help me. And that's it. They're reinforcing the (power) difference”. (LT3)

Teachers based in the UK talked about how the power gap narrows as students' progress through training, and how a wider gap is necessary and accepted by both parties during the early years.

“Certainly, second years will need you to be more distant because they really do just need you to tell them what they need to learn”. (LT3)

In contrast, some participants from Sri Lanka indicated that hierarchy extended from the culture of pre‐tertiary education, and was less amenable to change.

“In Sri Lanka most students [have] adapted to a system where they have to be guided by someone else from very younger age. [KS7]

“In our context, I see how we don't really move very far from the didactic power relationship … that distance among students and teachers is quite wide. It's essentially because of the culture that we have and the kind of upbringing that we have in this country.”. (KT3)

### 3.4 Narrowing the power distance in online learning

Participants in this study appeared to favour face‐to‐face learning, as a more familiar and “*personal*” setting in which “*mingling*” could support the building of rapport.

“It's the ability to socialize. And obviously also if we're in the building sometimes, if I'm sitting having a coffee …. Sometimes students will just come and chat informally. You know, and I think it's having that ability to build up those relationships in the whole …. A relationship in the round as opposed to a virtual relationship when you're both staring at your laptop or your computer”. (LT1)

“I can't see my lecturer [online]. It doesn't make it feel personal, so it makes the power difference feel larger because I feel like there's this all‐knowing voice (smiling), telling me things. But I can't put a name to a face. There's that loss of personal touch there”. (LS2)

**FIGURE 6:**
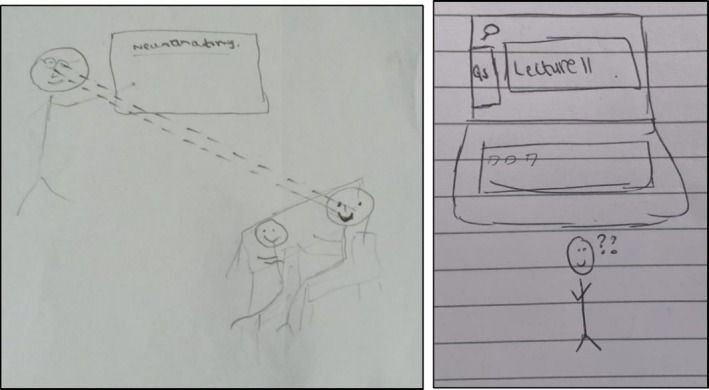
Establishing rapport in the face‐to‐face environment (KS3) and inability to do so online (LS2). [Color figure can be viewed at wileyonlinelibrary.com]

The face‐to‐face environment was deemed more conducive to building trust and relationships, through the ability to ‘see’ people, establish eye contact and observe non‐verbal communication (see Figure 6).

However, participants did recognise that there are features of online learning that can facilitate a reduction in power distance, in either favourable or problematic ways. These features included shared vulnerability, student autonomy and different communication possibilities.

#### 3.4.1 Shared vulnerability

When teachers were new to online facilitation, they could feel less powerful than in face‐to‐face settings.

“In the classroom I feel, probably more powerful because I can do my job better. Whereas online, I don't think I work as well, so maybe I don't feel as powerful”. (LT2)

“When I'm going to start a live online session, I feel like I'm just in front of a laptop, you know, just seeing at this screen, and I'm talking to my own computer”. (KT4)

However, there were indications that teachers' lack of familiarity with technology could promote a sense of shared vulnerability that helped foster mutual understanding. For example, both the teacher and the students being in their home environments, in casual clothes and facing the same technical issues helped students better relate to their teachers.

“I actually think …. the transaction of distance can be bridged much better with vulnerability and mutual experiences. We are all having trouble with Internet that is a shared experience. It makes us sympathize with each other and it builds some sort of bond”. (LT6)

#### 3.4.2 Student autonomy

Students in Sri Lanka reported that the online environment offered students greater power by allowing them to exercise control over their level of participation.

“In the online setting, the student actually has the power to do generally whatever he wants”. (KS11)

**FIGURE 7:**
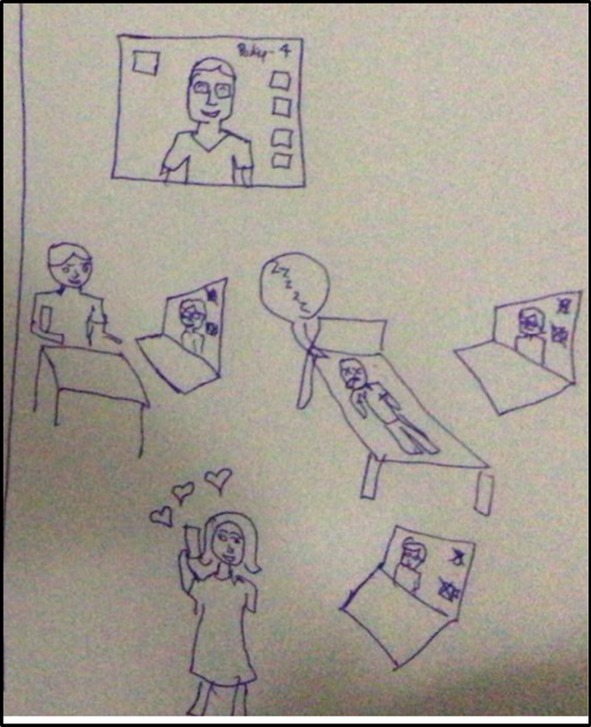
Varied student engagement online (KS7). [Color figure can be viewed at wileyonlinelibrary.com]

At the same time, they acknowledged that when teachers had less control, student engagement and as a result, learning could suffer (see Figure 7).

“As it is our decision to participate or not, most of the students don't participate”. (KS7)

#### 3.4.3 Different communication possibilities

Effective communication and its impact on learning were discussed widely. Regardless of the educational context, teachers' commitment to friendly and respectful communication was regarded as key in developing rapport and encouraging students to converse, thereby reducing the power gap. Addressing students as colleagues and using minimal technical language during teaching were strategies students felt reduced this distance.

Some teachers observed how students who were inhibited from communicating due to power distance, felt more empowered in doing so within the safety of the online environment. Furthermore, Sri Lankan students overcame barriers to communication they experienced in the physical space, as they were no longer expected to communicate through a class representative.

“Onsite … there's a huge gap between the lecturer and the students because if you have questions, we should go through the batch representatives. But in online learning, we could directly access the lecturer”. (KS5)

Though this quote highlights the potential for enhancing communication and reducing power distance through the use of online chat, one UK student acknowledged the limitations of online communication.

“In a lecture theatre, you're with your community of students, so you all feel like you're in the same boat. So if things are confusing, you can all kind of rally together to ask these questions … .. it's a confusing environment [online] and feels like there's a bigger sort of power distance because you're very alone in that situation”. (LS2)

“Chat access is quite limiting and it kind of increases the power distance … I have to then really think about how I'm phrasing my question because I'm using words and I'm using a limited sort of chat function to talk to my lecturer”. (LS2)

Some students also described situations where they were unable to grasp the material, and the teacher would continue with the lesson, unaware that students were struggling to follow (due to the lack of non‐verbal cues). Teachers supported this view, explaining how body language in face‐to‐face interactions, allowed them to identify struggling students. This was depicted through the use of bi‐directional arrows in drawings of the face‐to‐face environment as opposed to uni‐directional arrows in the online environment (Figure 1).

These findings suggest that online communication may be favourable to students, due to the ease of access to the teacher. However, some students might find this mode of communication quite complex and as a result could feel more alienated.

## 4 DISCUSSION

At the outset of this study, we expected to find differences between the Sri Lankan and UK contexts in terms of perceptions of power distance. Hofstede's 6‐D model of National Culture led us to assume that UK medical teachers and students would be less accepting of power distance in the student‐teacher relationship.^7^, ^8^ However, we found that teachers and students in both contexts expected and observed a degree of power distance between teachers and students, and employed strategies to exert or lessen power to influence learning. This section discusses these findings, before proposing recommendations for face‐to‐face and online teaching.

### 4.1 How is power understood within Sri Lankan and UK medical student‐teacher relationships?

In both Sri Lanka and the UK, the teacher's role was associated with authority and control. Drawings and interview responses conveyed that authority was needed to maintain student engagement and that traditional views of the teacher as a ‘sage on the stage’ persist.^4^ Perhaps traditional hierarchies within medicine are stronger than the national culture in guiding educational practices. Non‐clinical teachers perceived they were less powerful than clinical colleagues, and it may be that clinical teachers are expected to hold power due to their responsibility for patient safety or subject matter knowledge.^5^ Our interviewees suggested that in medicine, knowledge is power and both teachers and students expect and accept authority from senior clinicians.

However, teachers and students in both countries suggested that understandings of power are beginning to shift and there is a desire to flatten traditional hierarchies in medical education. Sri Lankan teachers reflected on the need to overcome students' histories of traditional student‐teacher relationships prior to medical school, whereas UK teachers referred to a more transactional University system and the need to interact with students as colleagues as they progressed towards clinical practice. Drawings and interview responses from both countries conveyed a wish for greater balance in power between teachers and students, though strategies for lessening power were somewhat limited to communication strategies and enabling students to ask questions.

Teachers in both countries suggested they conveyed greater vulnerability in online settings, but there was limited acknowledgement of the diverse knowledge and perspectives students can bring to learning. This may suggest a need for further promoting student‐centred and strengths‐based teaching approaches in medical education, in line with wider higher education developments.^29^

### 4.2 Does power distance between undergraduate medical students and teachers differ in the online and face‐to‐face learning environments? Do these perceptions vary between the contexts of Sri Lanka and the UK?

The participants in this study seemed to prefer face‐to‐face learning as a mode for facilitating rapport and trust, which they recognised as foundations for relationship‐building and shifting the balance of power.^30^ However, this was likely reflective of the timing of our study, when both teachers and students were adjusting to increased online learning resulting from the pandemic. It would be interesting to repeat this study now that online learning has become more commonplace and teachers and learners have become more digitally competent.

Although the online environment was considered by our participants to be isolating, they also recognised that it could offer a sense of safety, shared experience and vulnerability. Online learning presented opportunities in which students could exercise control and was valued for different communication possibilities, such as the potential for students to interact with the teacher through the chat function.^31^ There was limited recognition of the ways in which online learning could be enabling for students with illnesses or disabilities, or who have work or caring responsibilities. This may have been because participants were largely reflecting on synchronous interactive online learning experiences, rather than resources that could be viewed at the students' convenience.

In both cultural contexts and learning modes, it was clear that power was viewed as dynamic and teachers perceived they held the capacity to influence whether power enhanced or detracted from learning. Teachers in both Sri Lanka and the UK described adapting their verbal and body language and arrangement of educational spaces to promote students' participation in learning and provided helpful examples of the skills medical teachers have at their disposal to reduce power distance. These examples formed the basis of the recommendations below.

## 5 RECOMMENDATIONS FOR FACE‐TO‐FACE AND ONLINE LEARNING

Interview responses indicated it is important for the teacher to continually monitor and promote participation in learning, to confirm students' understanding, identify struggling students and ensure two‐way interaction.^32^, ^33^ Teachers must be particularly mindful that a variety of technical and non‐technical barriers can influence online engagement, and they must employ appropriate strategies to address these.^31^ Similarly, teachers can face many challenges in developing connections remotely and may require institutional support in overcoming them.^34^

Medical teachers should use power when a situation calls for it, such as when students stray off task or require redirection. However, abusing power, for example, through public humiliation, should always be avoided, given its far‐reaching negative effects on learning and wellbeing.^35^

To ensure a healthy power gap and thereby support learning, we recommend that teachers draw on the wide range of possibilities afforded by online and face‐to‐face environments. Strategies for reducing power distance include addressing students by name, respectful communication, establishing common ground, showing your ‘humane’ side, avoiding use of physical objects associated with power and arranging learning spaces appropriately. We also encourage teachers to consider ways they can acknowledge students' diverse knowledges and perspectives and invite student contributions to optimise everyone's learning.

## 6 STRENGTHS AND LIMITATIONS

The rich picture method was revealing in conveying teachers' and students' understandings of power in student‐teacher relationships, particularly as power is an intangible and complex concept. We found that basing discussions on drawings helped participants articulate their thoughts with relative ease, paving the way for rich and in‐depth exploration of perceptions. We strongly recommend this method to other educators who are studying complex issues close to their own practice. The adaptations we made to suit the remote nature of our study proved effective and could readily be adopted in other studies to employ the rich picture method online.

This study was undertaken during the Covid‐19 pandemic. This context led to some limitations in the study methods. Recruitment of students was difficult, perhaps due to the stressors of the pandemic and the need to prioritise learning that had been missed. We had assumed medical education students would be interested in participating, but were only able to recruit 2 students from Leeds (UK), despite reminder emails. It is possible we may have encountered more varied understandings of power distance had we recruited from a wider student pool.

A further limitation may have been that many of the teachers were relatively new adopters of online learning, meaning that online learning was often portrayed as a lesser teaching mode than face‐to‐face learning. Our study method of asking participants to draw two separate drawings of face‐to‐face and online learning may have further brought out this view. However, we were pleased to note that even new adopters were able to recognise the benefits of online learning related to reducing power distance.

## 7 CONCLUSIONS/IMPLICATIONS

In both Sri Lanka and the UK, power was understood as important for maintaining authority, student engagement and boundaries within the teacher‐student relationship. A degree of power distance was expected, though teachers and students expressed a desire for two‐way communication to enable clarification and challenge. Teachers attempted to lessen traditional hierarchies and promote participation, through strategies such as positioning, using student names and sharing of personal anecdotes.

Participants in this study seemed to favour face‐to‐face learning for relationship‐building, though recognised that online learning could afford different communication possibilities (e.g. online chat) and promote a sense of shared vulnerability. It may be that the sudden changes arising from the pandemic led to greater acceptance of humility and uncertainty, and it would be interesting to explore whether teachers have continued to reveal their ‘humane’ side as they have become more familiar with digital tools. It is recommended that teachers continue to attend to power distance in their relationships with students and to modulate power in the ways described in this paper. Regardless of whether teaching is online or face‐to‐face, we recommend recognising the power of partnership in learning and acknowledging students' diverse knowledge and perspectives.

## AUTHOR CONTRIBUTIONS

**Amaya Ellawala:** Conceptualization (lead); methodology (lead); investigation (equal); formal analysis (lead); writing—original draft (co‐lead); writing—review and editing (co‐lead). **Alison Ledger:** Conceptualization (supporting); formal analysis (supporting); writing—original draft (co‐lead), writing—review and editing (co‐lead). **Harith Wickramasekara:** Conceptualization (supporting); investigation (equal); formal analysis (supporting); writing—original draft (supporting), writing—review and editing (supporting).

## CONFLICT OF INTEREST STATEMENT

The authors have no conflicts of interest to disclose.

## Data Availability

Excerpts from the data are included in the paper for trustworthiness and transparency. We do not have ethical approval to share raw interview data, which may be identifiable.
